# A European Database of *Fusarium graminearum* and *F. culmorum* Trichothecene Genotypes

**DOI:** 10.3389/fmicb.2016.00406

**Published:** 2016-04-06

**Authors:** Matias Pasquali, Marco Beyer, Antonio Logrieco, Kris Audenaert, Virgilio Balmas, Ryan Basler, Anne-Laure Boutigny, Jana Chrpová, Elżbieta Czembor, Tatiana Gagkaeva, María T. González-Jaén, Ingerd S. Hofgaard, Nagehan D. Köycü, Lucien Hoffmann, Jelena Lević, Patricia Marin, Thomas Miedaner, Quirico Migheli, Antonio Moretti, Marina E. H. Müller, Françoise Munaut, Päivi Parikka, Marine Pallez-Barthel, Jonathan Piec, Jonathan Scauflaire, Barbara Scherm, Slavica Stanković, Ulf Thrane, Silvio Uhlig, Adriaan Vanheule, Tapani Yli-Mattila, Susanne Vogelgsang

**Affiliations:** ^1^Department of Environmental Research and Innovation, Luxembourg Institute of Science and TechnologyBelvaux, Luxembourg; ^2^Institute of Sciences of Food Production, National Research CouncilBari, Italy; ^3^Department of Applied Biosciences, Faculty of Bioscience Engineering, Ghent UniversityGhent, Belgium; ^4^Department of Agriculture, University of SassariSassari, Italy; ^5^BIOGER UMR, INRAThiverval-Grignon, France; ^6^ANSES, Plant Health LaboratoryAngers, France; ^7^Division of Crop Genetics and Breeding, Crop Research InstitutePrague, Czech Republic; ^8^Department of Grasses, Legumes and Energy Plants, Plant Breeding and Acclimatization Institute-National Research InstituteRadzikow, Poland; ^9^Laboratory of Mycology and Phytopathology, All-Russian Institute of Plant ProtectionSt. Petersburg, Russia; ^10^Department of Genetics, Faculty of Biology, Complutense University of MadridMadrid, Spain; ^11^Norwegian Institute of Bioeconomy ResearchÅs, Norway; ^12^Department of Plant Protection, Agriculture Faculty, Namık Kemal UniversityTekirdag, Turkey; ^13^Laboratory of Phytopathology and Entomology, Maize Research Institute Zemun PoljeBelgrade, Serbia; ^14^Plant Breeding Institute, University of HohenheimStuttgart, Germany; ^15^Leibniz Centre for Agricultural Landscape Research, Institute for Landscape BiogeochemistryMüncheberg, Germany; ^16^Applied Microbiology, Earth and Life Institute, Université Catholique de LouvainLouvain-la-Neuve, Belgium; ^17^Department Natural Resources and Bioproduction, Natural Resources Institute Finland (Luke)Jokioinen, Finland; ^18^Section for Eukaryotic Biotechnology, DTU Systems Biology, Technical University of DenmarkKongens Lyngby, Denmark; ^19^Section for Chemistry and Toxicology, Norwegian Veterinary InstituteOslo, Norway; ^20^Molecular Plant Biology, Department of Biochemistry, University of TurkuTurku, Finland; ^21^Research Division Grassland Sciences and Agro-Ecosystems, Institute for Sustainability Sciences, AgroscopeZürich, Switzerland

**Keywords:** acetyldeoxynivalenol, chemotype, database, *Fusarium*, genotype, mycotoxin, nivalenol, trichothecene

## Abstract

*Fusarium* species, particularly *Fusarium graminearum* and *F. culmorum*, are the main cause of trichothecene type B contamination in cereals. Data on the distribution of *Fusarium* trichothecene genotypes in cereals in Europe are scattered in time and space. Furthermore, a common core set of related variables (sampling method, host cultivar, previous crop, etc.) that would allow more effective analysis of factors influencing the spatial and temporal population distribution, is lacking. Consequently, based on the available data, it is difficult to identify factors influencing chemotype distribution and spread at the European level. Here we describe the results of a collaborative integrated work which aims (1) to characterize the trichothecene genotypes of strains from three *Fusarium* species, collected over the period 2000–2013 and (2) to enhance the standardization of epidemiological data collection. Information on host plant, country of origin, sampling location, year of sampling and previous crop of 1147 *F. graminearum*, 479 *F. culmorum*, and 3 *F. cortaderiae* strains obtained from 17 European countries was compiled and a map of trichothecene type B genotype distribution was plotted for each species. All information on the strains was collected in a freely accessible and updatable database (www.catalogueeu.luxmcc.lu), which will serve as a starting point for epidemiological analysis of potential spatial and temporal trichothecene genotype shifts in Europe. The analysis of the currently available European dataset showed that in *F. graminearum*, the predominant genotype was 15-acetyldeoxynivalenol (15-ADON) (82.9%), followed by 3-acetyldeoxynivalenol (3-ADON) (13.6%), and nivalenol (NIV) (3.5%). In *F. culmorum*, the prevalent genotype was 3-ADON (59.9%), while the NIV genotype accounted for the remaining 40.1%. Both, geographical and temporal patterns of trichothecene genotypes distribution were identified.

## Introduction

Fusarium head blight (FHB) is one of the most important cereal diseases worldwide. Severe outbreaks of FHB may result in significant yield losses of up to 50%, depending on the small grain cereal crop (Parry et al., 1995). McMullen et al. (2012) suggested that FHB in the United States might lead to economic losses in excess of one billion USD per year. More importantly is the production of secondary metabolites, specifically mycotoxins, contaminating the harvested products and thus jeopardizing food and feed safety (e.g., Snijders, 1990).

In cereals, FHB is usually caused by a set of different *Fusarium* species, with different life styles and different types of mycotoxins produced. Within the *Fusarium graminearum* species complex (FGSC; O'Donnell et al., 2000), which presently includes 16 species (Aoki et al., 2012), *F. culmorum* and *F. cerealis* are among the most dominant pathogens causing head blight on wheat and other cereals worldwide (Moss and Thrane, 2004; Osborne and Stein, 2007). Other frequently detected species are *F. poae, F. avenaceum, F. langsethiae, F. tricinctum, F. sporotrichioides* (Ioos et al., 2004; Xu et al., 2005; Xu and Nicholson, 2009; Somma et al., 2014), and the non-toxigenic species *Microdochium nivale* and *M. majus* (Glynn et al., 2005).

One of the main *Fusarium* mycotoxin classes are the trichothecenes, sesquiterpene epoxides that inhibit eukaryotic protein synthesis, which may cause severe toxicosis in humans and animals (Ueno, 1983; Maresca, 2013). *Fusarium* trichothecenes are grouped into two classes based on the presence (type B) vs. absence (type A) of a keto group at the C-8 position (Kristensen et al., 2005). Depending on differences in the core trichothecene cluster (*TRI* cluster), which includes two regulatory genes (*TRI*6 and *TRI10*) and most of the biosynthetic enzymes required for the production of trichothecenes (Kimura et al., 2007; Alexander et al., 2011), *Fusarium* species as well as individual strains may produce different types of trichothecenes.

Among the type B trichothecenes, the following are considered to have a significant impact on food and feed safety: deoxynivalenol (DON), nivalenol (NIV), and their acetylated derivatives 3-acetyldeoxynivalenol (3-ADON), 15-acetyldeoxynivalenol (15-ADON), and 4-acetylnivalenol (4-ANIV, syn. fusarenone-X; Eriksen et al., 2004; Desjardins, 2006).

Different *Fusarium* species chemotypes have been described: chemotype I, produces DON and/or its acetylated derivatives while chemotype II, produces NIV and/or 4-ANIV (Sydenham et al., 1991). The DON chemotype can be further split into chemotype IA (producing 3-ADON) and IB (producing 15-ADON; Miller et al., 1991). Structural differences among toxins from different chemotypes may have consequences on strain fitness, since the specific pattern of oxygenation and acetylation can modify the bioactivity and hence the (phyto) toxicity of these compounds (e.g. Ward et al., 2002; Alexander et al., 2011). As it has been shown in a large survey on Canadian grains, DON and NIV, being the two most abundant toxins detected, now represent the two major concerns for safety of wheat and barley products (Tittlemier et al., 2013).

Environmental factors may result in a geographical partitioning of subpopulations that may coincide with chemotypes. The success of a given chemotype, which is of importance with respect to FHB control, is often related to local factors (van der Lee et al., 2015). Based on chemotype characterization of Italian *Fusarium* species, Covarelli et al. (2015) suggested that climatic conditions have a strong impact on the occurrence of 3-ADON and 15-ADON whereas NIV contamination occurred irrespective of climatic conditions. Yli-Mattila et al. (2013) proposed that the prevalence of a specific chemotype may also be influenced by a certain host. For example, NIV-producing strains were found to be more aggressive towards maize compared with DON-producers (Carter et al., 2002) and were associated, in *F. asiaticum*, preferentially to maize in China (Ndoye et al., 2012). Maier et al. (2006) postulated NIV to be a virulence factor in maize, which is in line with findings that associate an increase in NIV populations in areas where the preceding crop was maize (Audenaert et al., 2009; Pasquali et al., 2010; Sampietro et al., 2011).

Two main reasons to determine the chemotype of a strain have been proposed (Pasquali and Migheli, 2014): (1) to obtain epidemiological information on the population colonizing a crop in a given area, using chemotype as a proxy in the field; (2) to inform on the toxigenic risk of contaminated food or feed determined by the presence of a certain chemotype, with the long term perspective of developing preventive models and strategies to decrease the risk.

At present, data on chemotype distribution of FGSC are available from all continents (reviewed in Pasquali and Migheli, 2014), being *F. graminearum sensu stricto* (s.s.) the most studied species. Less work has been devoted to chemotype determination in *F. culmorum* (Scherm et al., 2013). Shifts in species population have been reported in many surveys (Xu et al., 2005; Nielsen et al., 2011; Fredlund et al., 2013), but reports on chemotype shifts in certain areas are more recent (e.g. Nielsen et al., 2012; Beyer et al., 2014).

Despite the fact that information from all continents is now available, most reports do not include complete information on the strains analyzed, such as geographic origin, host from which it was isolated, method used for species identification, etc. In addition, precise characterization of the species is frequently lacking, being based only on morphological observations, hence, making it unfeasible to use the dataset for further studies.

The main goal of this joint study was to generate an accessible map of trichothecene genotypes from three FHB causing species with detailed information on how the data were obtained. This will allow, in the long term, the acquisition of consistent and homogenous datasets providing a valid comparison of the distribution of chemotypes during years and among countries. To reach this aim, research institutions from 17 European countries were inquired to provide data on how the sampling was performed as well as detailed information on cropping history and location. A more coordinated effort, leading to common protocols for sampling, chemotype determining and data reporting in a more accessible way would increase the standardization of epidemiological data. Furthermore, it could facilitate the effort of understanding which factors do favor establishment and persistence of a specific chemotype. This collective effort is now assembled in a fully accessible and upgradable dataset of chemotype diversity within FGSG and *F. culmorum* on cereals in Europe.

## Materials and methods

### Data collection

An Excel template file was sent to research partners agreeing to participate in the initiative (Supplementary File 1). The information to be submitted (if applicable) were as follows: chemotype, year of isolation, whether the strain was obtained by a single spore or a single hyphae, the location including the geographic coordinates, the crop host and cultivar from which it was isolated, previous crop, method of isolation, method used for species attribution, primers and/or gene in case of PCR and sequencing, name of culture collection in case it was deposited, strain ID, and citation of the strain/s in a publication. Whenever genetic chemotype or species was unknown, strains were shipped to the Luxembourg Institute of Science and Technology (LIST) laboratory for genetic chemotyping (Pasquali et al., 2011) and species identification by sequencing EF1alpha (Geiser et al., 2004). The overall dataset (www.catalogueeu.luxmcc.lu; available as of mid-April 2016) was built through integrating data communicated by research partners and by laboratory results obtained with the procedures described below.

### DNA extraction and chemotype determination

Fungal colonies were grown on PDA as described in Pallez et al. (2014) in order to directly extract DNA using a rapid procedure. Briefly, a 2–5 mm piece of miracloth tissue (Millipore, USA) covered by a 5 days old fungal culture, was collected and added to 100 μL TE (10 mM Tris-Cl, 0.05 mM pH 9 EDTA solution, Sigma-Aldrich, USA). After 5 min of microwave treatment at 900 W and a 30 s centrifugation at 13,000 g, 5 μL were then used for PCR reactions. When identification of the strain was reported to be putative by partners, EF1alpha amplification was carried out, followed by sequencing as described in Dubos et al. (2011). If the species was previously defined, tri12 multiplex PCR for genetic chemotyping (Ward et al., 2002) was carried out. All PCR reactions were performed in a 50 μL volume to avoid risk of PCR inhibition due to the quick extraction method using 2X Phusion master mix (Thermo, USA), 300 nM of each primer and water. Thermal cyclers used were Biometra T-professional and Veriti PCR Thermal cyclers (Life Technologies, USA) using the programme as described in Pasquali et al. (2011). All reactions included positive controls for the three chemotypes and a negative control for monitoring potential contaminations. Reactions were visualized on a Biorad agarose ready to use gel at 3%, using a UV spectra analyzer (Ingenius, Syngene, UK). When results were ambiguous the reaction was repeated at least once.

Data assembled from other laboratories were collected by the Excel template file and uploaded to the database page. When diverse methods for genetic chemotyping were used (Waalwijk et al., 2003; Jennings et al., 2004; Quarta et al., 2006; Starkey et al., 2007; Yli-Mattila and Gagkaeva, 2010) by the original isolating laboratory, this fact is specified directly in the database.

### Statistical analysis and visualization tools

Descriptive graphs on species and chemotype distribution were obtained using SigmaPlot version 12.5 **(**Systat Software Inc, USA) and SPSS version 19 (IBM, USA). The European maps generated for this study were prepared using the ArcMap platform (ESRI Inc., USA). A Multiple Correspondence Analysis tool (Broeksema et al., 2013) was used for studying the overall dataset and its homogeneity in relation to species and chemotype distribution. Logistic regressions were performed using SigmaPlot 12.5.

### Database construction

The European database was constructed by assembling the overall dataset on the database architecture developed by Piec et al. (2016). A filtering option for country and laboratory, the option to upload new datasets, with administrator validation, was added to the existing architecture. Functions of the database include the possibility to have a full or filtered download of the overall dataset.

## Results

The current work represents the first collective attempt to compile information on chemotype diversity occurring in European countries. Moreover, the availability of a full open access database provides for the first time a centralized source of information for *Fusarium* disease records on cereals, which is of high value for researchers working in the mycotoxin/*Fusarium* biodiversity domain.

### The database

**Figure 1:**
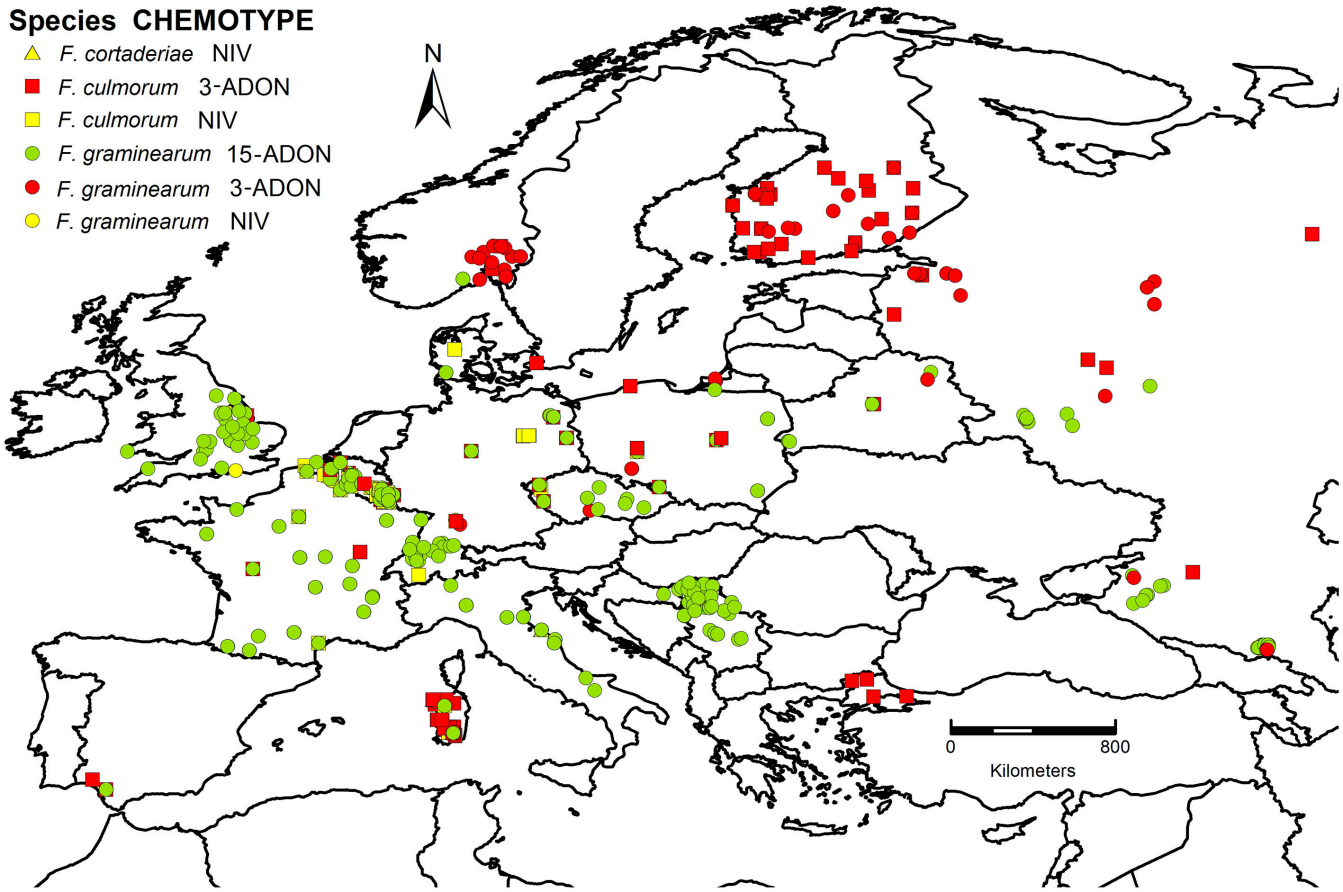
**Spatial distribution of chemotypes and *Fusarium* species in Europe**. 3-ADON, 3-acetyldeoxynivalenol; 15-ADON, 15-acetyldeoxynivalenol; NIV, nivalenol. *F. cortaderiae* were isolated in Italy but cannot be visualized as they are overlapped by other strains.

The overall dataset including all information collected for this work has been assembled in a database. Based on the previous architecture constructed for the LIST culture collection (Piec et al., 2016), a database with improved functionalities was built. The overall map with overlapping *F. graminearum* and *F. culmorum* species is shown on the first page of the database (www.catalogueeu.luxmcc.lu; Figure 1). Further uploading of data can be performed according to the instructions in Supplementary File 2. Researchers working on *Fusarium* toxigenic diversity are invited to contribute to the database or to download the dataset for further analysis.

### Data description

**Figure 2:**
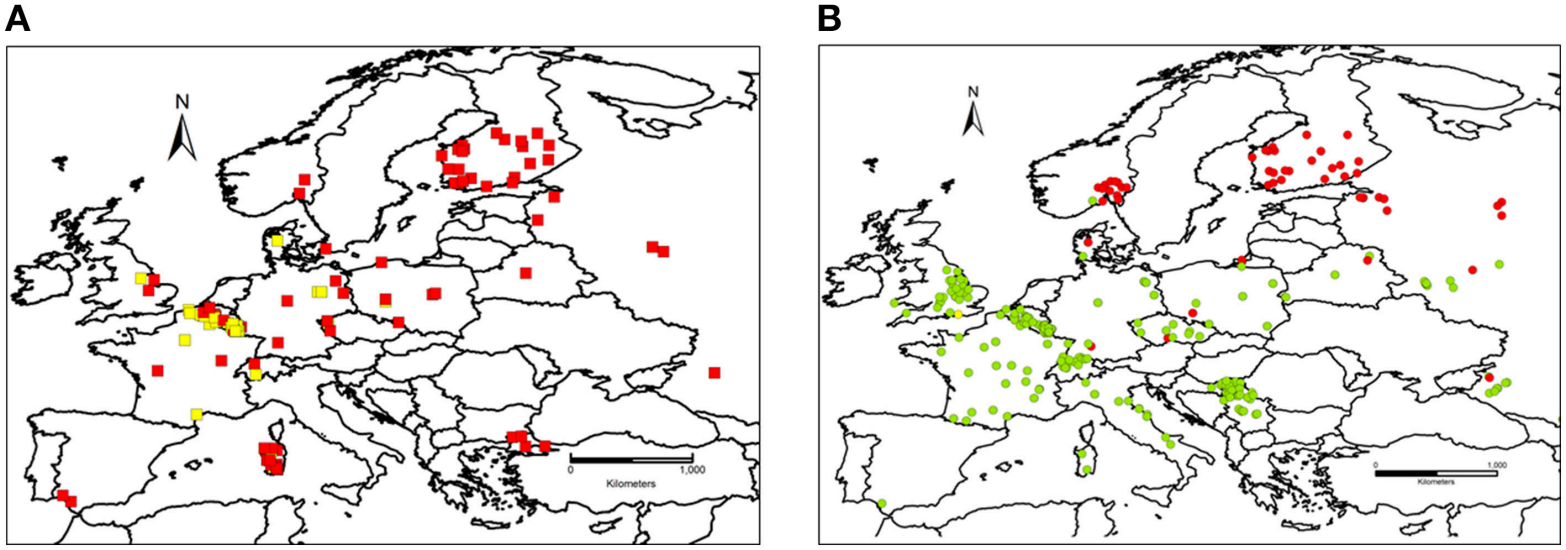
**(A) Spatial distribution of *Fusarium culmorum* chemotypes in Europe**. Red squares, genetic 3-ADON chemotype. Yellow squares, genetic NIV chemotype. **(B) Spatial distribution of *Fusarium graminearum sensu stricto* chemotypes in Europe**. Green circles, genetic 15-ADON chemotype. Red circles, genetic 3-ADON chemotype. Yellow circles, genetic NIV chemotype.

**Figure 3:**
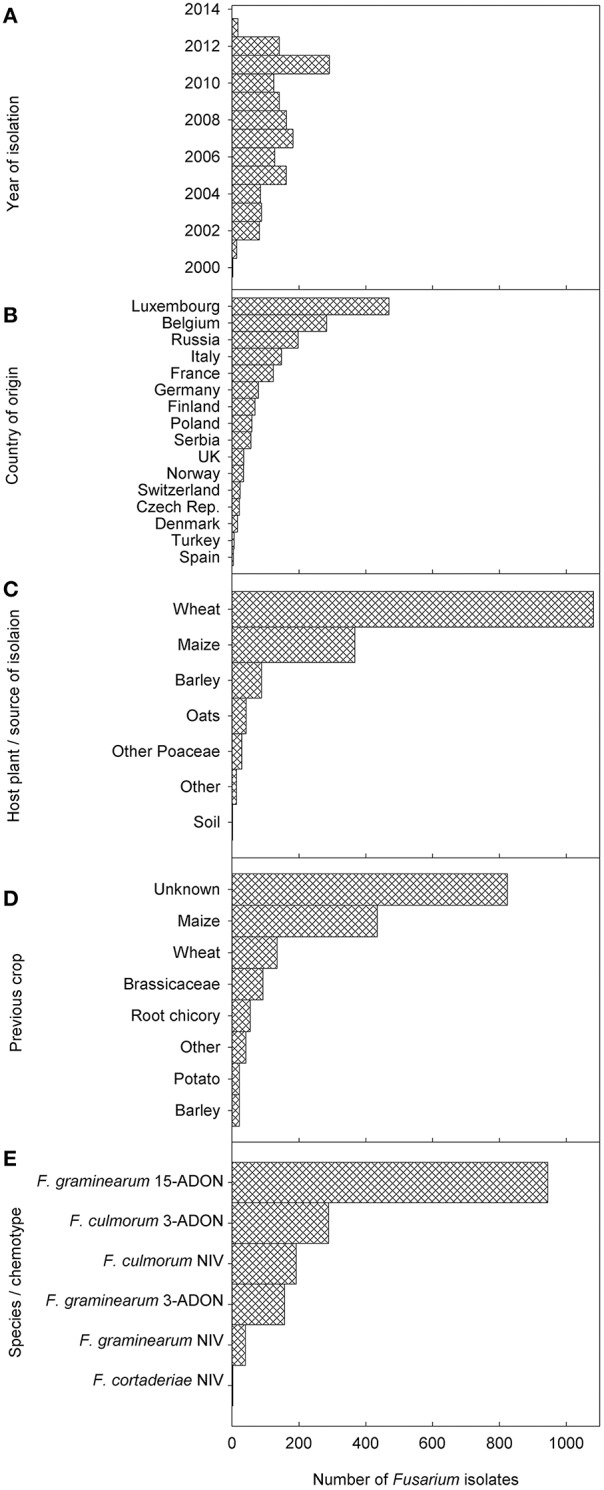
**Frequency distributions of (A) the years, when the fungal strains for the current database were isolated, (B) the countries of origin of the strains, (C) the host plants from which strains were isolated, (D) the previous crops (“Other” include mixtures of legumes and cereals, lucerne, lupines, perennial forages, spinach, and sugar beet), and (E) the species/chemotype combinations found**. Only 3 *Fusarium cortaderiae* strains were included and are thus not visible in this figure. In the host plant figure, “other crops” include thistles, soya and potatoes. “Other poaceae” include forage grasses, einkorn wheat, triticale, wild type barley.

Information of a total of 1147 *F. graminearum* and 479 *F. culmorum* strains was included in the dataset collected from the period 2000–2013 and plotted on the respective maps (Figures 2A,B). Years of isolation were close to homogeneity (Figure 3A). Luxembourg was the country where most strains were obtained, followed by Belgium and Russia (Figure 3B). At present, chemotype information from some countries is missing in the current dataset, therefore, further uploading of information will be important to obtain a more precise picture of chemotype distributions in Europe.

**Figure 4:**
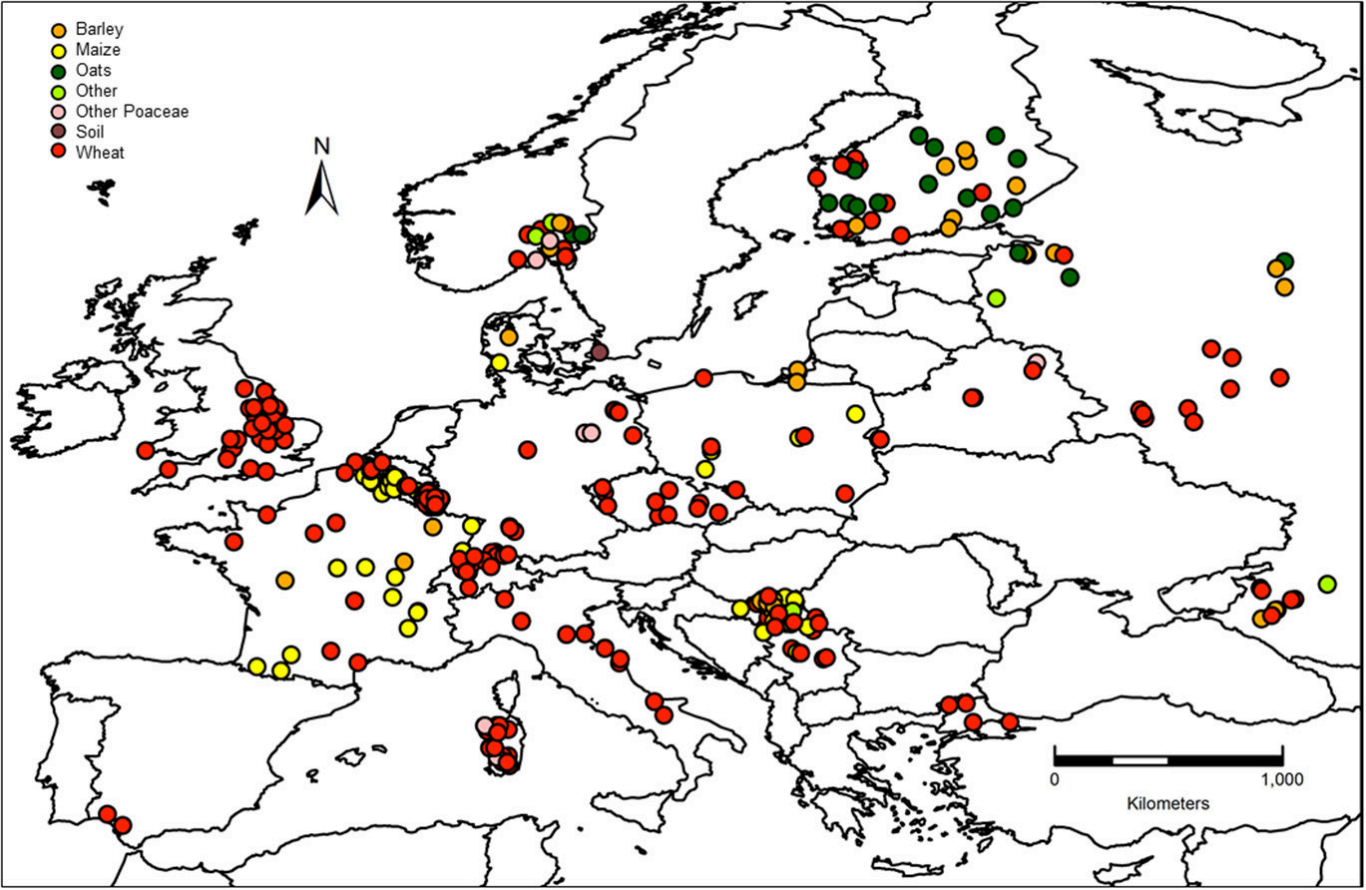
**Materials from which the fungal strains were isolated**.

The major crop from where strains were isolated was wheat (66.7%) followed by maize (22.5%), barley (5.4%), and other crops (combined 5.3%; Figure 3C). As can be observed by the map of crop distribution, wheat was sampled in 16 out of the 17 countries, whereas other crops were sampled in a limited number of countries (maize *n* = 6; barley *n* = 7; oats = 3; Figure 4). Oats were sampled only in Northern Europe, including Norway, Finland and Russia, where oats are an important crop, while no maize was sampled in Northern Europe, where the climate is not yet suitable for maize production.

Previous crop information was available only for a minority of samples (<50%). Therefore, the analysis of these data was postponed until enough data are uploaded to allow meaningful conclusions (Figure 3D).

With respect to the overall distribution of chemotypes per species for *F. graminearum*, the predominant genotype was 15-ADON (82.9%, 951 strains), followed by 3-ADON (13.6%, 156 strains), and NIV (3.5%, 40 strains). The 15-ADON genotype was most common in isolates from wheat and maize, while the 3-ADON genotype was most common in northern Europe and oats. For *F. culmorum*, the prevalent genotype was 3-ADON (59.9%, 287 strains), while the NIV genotype accounted for the remaining 40.1% (192 strains). Three *F. cortaderiae* with the NIV chemotype were also included (Figure 3E). The chemotype distribution within each country can be accessed through the filtering options available online (see Supplementary File 3 for graphical representation). Interestingly, only 3-ADON isolates of *F. graminearum* and *F. culmorum* were found in the collected European isolates from oats.

### Data analysis

**Figure 5:**
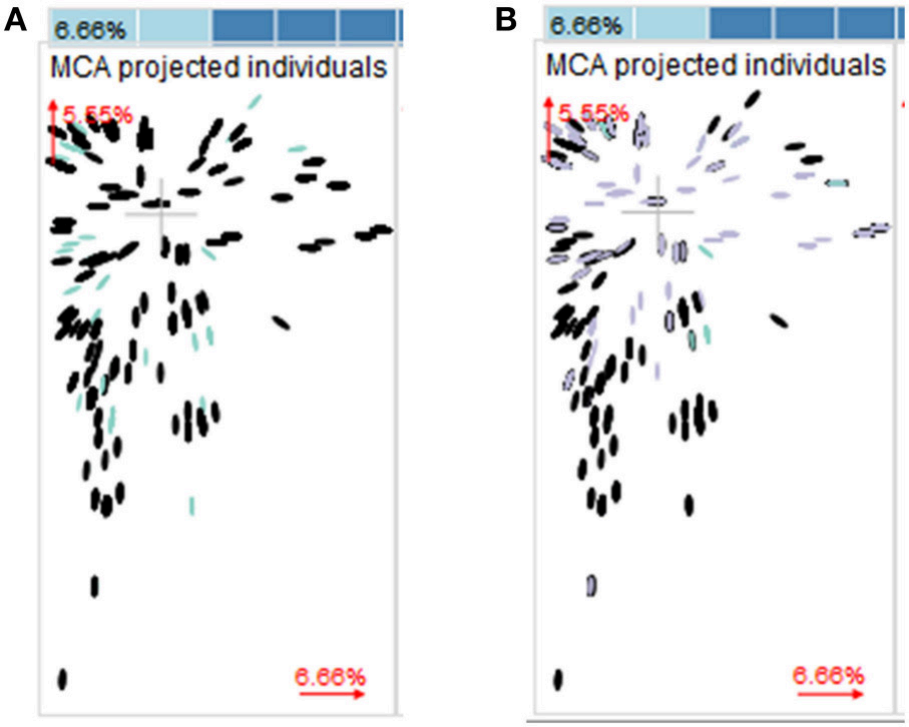
**Multiple correspondence analysis (MCA) with year, country, crop**. The two major components are plotted. Dimension 1 accounted for 6.66% of the variance in the data and dimension 2 accounted for 5.55% of the variance. Both dimensions reflect levels of association between the categories of the factors rather than the factors themselves. Groups of objects being close to each other share many properties, while objects (in our cases *Fusarium* strains) being distant, do not share many properties. If distinct groups occur in the plot, the data set contains enough information to separate the groups based on the categorical data that entered the MCA. If all groups are intermixed, the variables that entered the MCA are not suitable to distinguish the groups. **(A)** Colors indicate different *Fusarium* species (black, *Fusarium graminearum*; blue, *Fusarium culmorum*). **(B)** Colors indicate different chemotypes (black, 3-ADON; blue, NIV*;* violet, 15-ADON).

Multiple correspondence analyses including year, country, and host plant showed no evidence for a preferential association of the species (*F. graminearum* or *F. culmorum*) with specific countries (both species were present in 16 out of 17 countries), sets of countries, years or crops (Figure 5A). On the contrary, when the corresponding analysis was performed on the chemotype dataset, it was evident that chemotypes were not randomly distributed over countries, years, and crops (Figure 5B).

**Figure 6:**
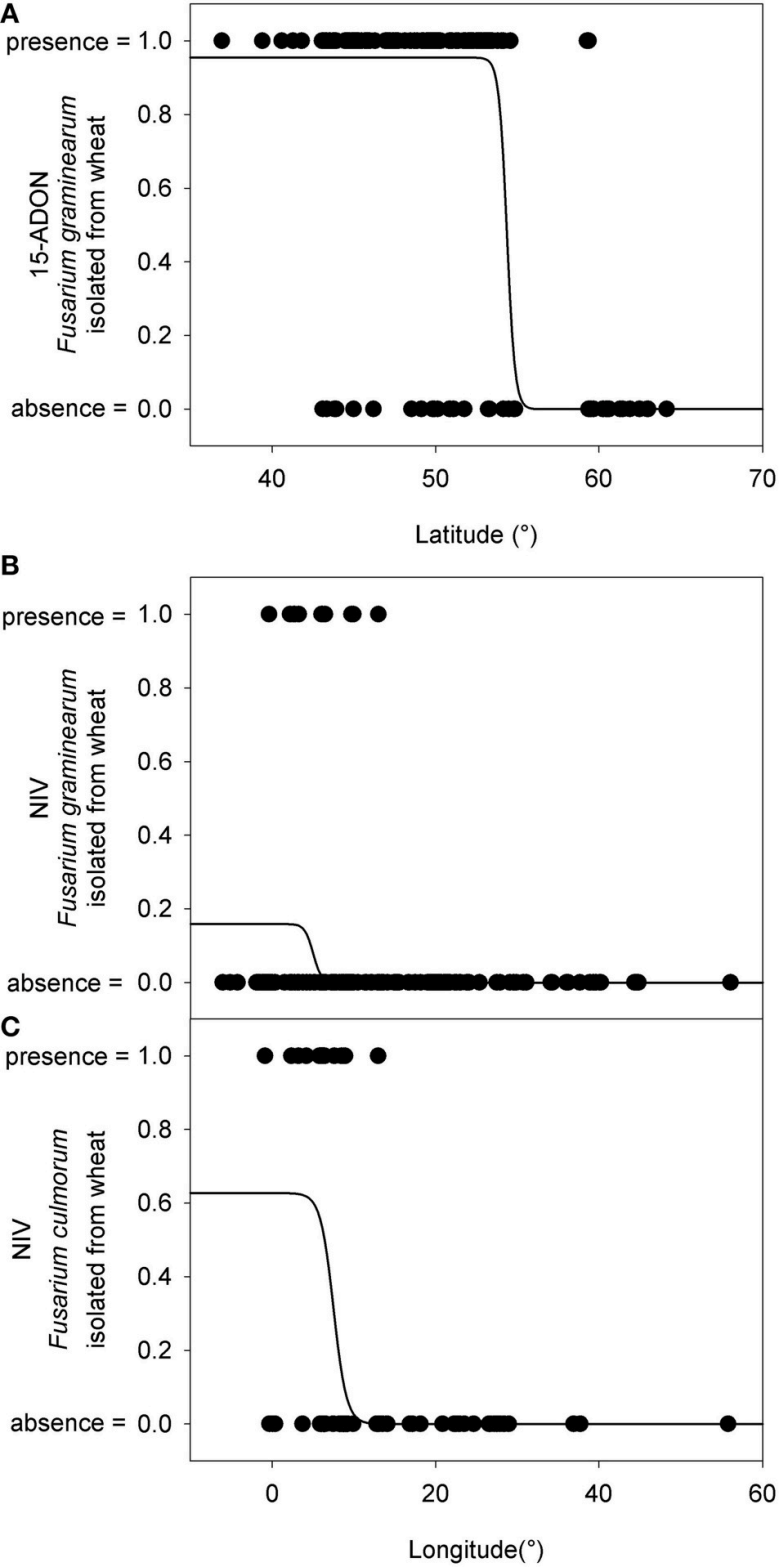
**Presence (=1) and absence (=0) of different chemotype strains within the European *Fusarium graminearum sensu stricto* and *F. culmorum* isolate collection in relation to the latitude where they were found. (A)** 15-ADON chemotype strains in *F. graminearum*. **(B)** NIV chemotype strains in *F. graminearum*. **(C)** NIV chemotype strains in *F. culmorum*. The logistic regression line gives an estimate about the probability of finding a particular isolate within the *F. graminearum*/*F. culmorum* population.

For further analyses of the chemotype distribution, we focused on the most abundant population obtained from the same host. A total of 784 *F. graminearum* s.s. strains were isolated from wheat. The 15-ADON chemotype was rarely observed in Northern Europe (Figure 2B). The latitude that marked the Northern limit of the 15-ADON chemotype distribution in *F. graminearum* strains isolated from wheat in Europe was estimated by logistic regression: Hardly any 15-ADON chemotype strains of *F. graminearum* were found above 54.4 ± 10.8° Northern latitude while the probability for a 15-ADON chemotype strain in a *F. graminearum* population more southwards converged to 95.5 ± 0.85% (Figure 6A).

Similarly, in *F. culmorum*, the percentage of NIV chemotype strains drastically dropped off East of 7.5 ± 0.6° longitude (Figures 2A,6C). West of 7.5 ± 0.6° longitude, the probability of observing a NIV chemotype strain in a *F. culmorum* population isolated from wheat converged to 62.7 ± 12.6%. In *F. graminearum*, the probability of finding a NIV chemotype strain was 15.9 ± 2.5% West of 5.0 ± 0.8° longitude East, but dropped quickly further eastwards Figures 2B,6B.

The availability of temporal series of strains allowed also verifying the possible shift of species or chemotypes in regions with high data density. By selecting the densest sampled area from the available dataset (Luxembourg, year 2007–2012), it was possible to observe how the NIV chemotype in *F. graminearum* disappeared at the end of the sampling period (Supplementary Video 1).

## Discussion

By assembling the dataset described here, we could establish a comprehensive collection of European data on *Fusarium* diversity on cereals. The analysis of the distribution of genetically determined chemotypes confirms the dominance of the 15-ADON chemotype in Western, Southern and Central Europe in *F. graminearum* (Pasquali and Migheli, 2014) but at the same time, we identified some current geographic limits to its distribution in wheat. Whether this depends on cropping and/or climatic factors merits further investigation given the fact that the 15-ADON chemotype is currently the major cause of DON accumulation in European wheat. Our data are generally in agreement with earlier reports on heterogeneous chemotype distribution in Europe (Yli-Mattila et al., 2009; Aamot et al., 2015; van der Lee et al., 2015). However, earlier reports did not estimate where the geographical limits of the chemotype spatial distribution are. Our study indicates a limited spread of 15-ADON trichothecene genotypes toward the Northern latitudes. This is confirmed by the results from a recent study in Norway where 3-ADON was the dominating trichothecene genotype (Aamot et al., 2015). However, the 15-ADON type was recently introduced into Norway, probably from other parts of Europe (Aamot et al., 2015), which exemplifies the need for a common database to monitor genotype shifts in Europe. A hypothesis worth testing would be to verify if parameters such as temperature, light irradiation, cropping practices, and/or host plants typical of certain latitudes may have an impact on the spread of the 15-ADON population by combining phenotypic tests with studies on the homogeneity of the fungal population.

The dominance of the 3-ADON chemotype in Northern Europe, which is in accordance with previous results of Yli-Mattila et al. (2009), Yli-Mattila and Gagkaeva (2010), and Yli-Mattila et al. (2013), was also confirmed. The role of oats as the potential preferential host for the 3-ADON population of *F. graminearum* seems to be confirmed in our dataset.

We also observed that the NIV chemotype was preferentially found in Western Europe. The fact that NIV chemotypes in *F. culmorum* were rarely found in Eastern locations, suggests that possibly different populations associated to chemotype diversity might be adapted to distinct cropping practices or to distinct climatic conditions, hence, further diversity studies on *F. culmorum* populations are needed.

Furthermore, we could identify temporal patterns of chemotype distribution that were partially associated to temporal changes in climatic conditions as observed in Luxembourg (Beyer et al., 2014). Enlarging the dataset would allow similar comparisons across European countries as has been done for example in North America and within China. In fact, analyses of the trichothecene chemotype distributions across Canada (Ward et al., 2008) revealed a dramatic longitudinal cline in which 3-ADON producers from wheat were significantly more common in Eastern Canada than in Western provinces (Ward et al., 2008), amounting to a 14-fold increase between 1998 and 2004. The authors suggested that the rapid increase in 3-ADON frequency in Western Canada might indicate that 3-ADON populations have a selective advantage over isolates from the resident 15-ADON population. The reason for the observed shift is unclear but they hypothesized that it could be due to changes in agricultural practices or environmental conditions. Guo et al. (2008) hypothesized that the shift occurring in Manitoba might have been produced by seed shipment and long distance transportation of spores. In the study by Zhang et al. (2010), where more than 400 isolates of *F. asiaticum* (part of the *F. graminearum* species complex) from barley at 18 sites (10–2000 km apart) in three valleys of Southern China were analyzed, a significant cline of 3-ADON producers was observed in the middle valley, but no correlations with climate or agronomy factors were identified.

Certainly, the dataset presented here includes some gaps that should be filled in order to perform increasingly reliable analyses on the potential causes of change in species or chemotype distribution. It is expected that further updates which could include also sowing date, tillage regime, fungicide treatments, and other cultural practices will strengthen the dataset and allow a better understanding of the effects of cropping measures and environmental factors on strain distribution.

Nevertheless, it is evident that molecular genotyping is a powerful tool to support or refute epidemiologically generated hypotheses (Litvintseva et al., 2015). Numerous databases related to fungal diversity and bioproducts are available (Wackett, 2015), as well as fungal repositories for specimens (Abd-Elsalam et al., 2010), but they rarely integrate different sources of information on strain diversity. Our database includes molecular datasets associated to geographic and cropping practices and with the availability of GPS data, our database can be indirectly enriched with meteorological datasets related to the location where the strains were collected.

It has been suggested that “large datasets containing epidemiological data associated to genetic information can help understanding, recognizing and eventually, managing fungal outbreaks” (Litvintseva et al., 2015). Hence, given the importance of shared data for fungal epidemiological studies and considering the interactions between environmental, cropping, and genetic factors, our up-scalable and fully open access database can possibly help addressing future risks of spread of toxigenic *Fusarium* populations on cereals.

This work also represents an example of a European participative and cooperative approach that can serve as an example for the establishment of other epidemiological studies profiting from the availability of large and well maintained datasets. Given the complexity of FGSC, future efforts will not only aim at inclusion of data from other countries and new fungal strains but will also have to integrate multi locus sequence genotyping information (MLST) to better characterize diversity and possibly even new species or the level of population diversity (Ward et al., 2002; Talas et al., 2011; Aamot et al., 2015; Talas and McDonald, 2015).

At the same time, this entirely accessible dataset is essential for allowing further targeted studies in order to fully differentiate all included strains at both species and subpopulation levels, assuming that *F. graminearum* as well as *F. culmorum* are constituted by different subpopulations (Liang et al., 2014; Balmas et al., 2015; van der Lee et al., 2015).

In the current version of the dataset, genetic determination of the chemotype was carried out to differentiate the three major known chemotypes in Europe. It is foreseeable to further analyze the strains by investigating the presence of the NX-2 chemotype that has been currently found only in the USA (Fruhmann et al., 2014; Liang et al., 2014; Varga et al., 2015).

Employing a flexible, up-scalable and upgradable database, our work represents the first attempt to build a global database in which strains, provided with information of GPS data and host, are also analyzed using multi-locus genotyping in combination with VNTR (Variable Number Tandem Repeat) screening and potentially whole genome sequencing (van der Lee et al., 2015). The success of such an effort will depend on the future contributions: the availability of a well-maintained and expanded database is a solid contribution to a shared approach philosophy of conducting research that will help to speed up scientific progress in fungal biology and agriculture (Abbà et al., 2015).

## Author contributions

MP conceived and performed the experiments, coordinated the assembly, analyzed the data, and wrote the manuscript. SV conceived the experiments, coordinated the assembly, assembled and analyzed the data, and wrote the manuscript. MB performed the experiments, analyzed the data and wrote the manuscript. JP assembled and managed the database. AL, KA, VB, RB, AB, JC, EC, TG, MG, IH, NK, LH, JL, PG, TM, QM, AM, MM, FM, PP, MP-B, JS, BS, SS, UT, SU, AV, and TY performed experiments and helped in writing the manuscript.

## Funding

The Luxembourg Institute of Science and Technology, LU, acknowledges the Ministère de l'Agriculture, de la Viticulture et de la Protection des Consommateurs-Administration des Services Techniques de l'Agriculture for financially supporting the Sentinelle project. The work on Italian strains has been financially supported through the M.I.U.R. Project AGROGEN (Laboratory of GENomics for traits of AGROnomic importance in durum wheat: Identification of useful genes, functional analysis and assisted selection by biological markers for the development of the national seed chain) (D. D. 14.03.2005 n. 602/Ric). Funding for the research of Ryan Basler was provided by Felix Thornley Cobbold Trust and the John Oldacre Foundation.

The work of JC was supported by the Ministry of Agriculture of the Czech Republic, Project No. RO0415. The research of MG and PG was supported by the Spanish Ministry MINECO (AGL2014-53928-C2-2-R). The Ministry of Agriculture and Food, Norway funded the work of IH. The research of TM was funded by the Federal Ministry of Education and Research (BMBF) (GABI-KANADA #FKZ 0313711A), Bonn and by the German Academic Exchange Service (DAAD), Bonn (code no.: A/06/92183). PP acknowledges the Finnish Ministry of Agriculture and Forestry for funding the project FinMyco on *Fusarium* and mycotoxins in Finland. The research of JS was funded by the Direction Générale de l'Agriculture, Direction de la Recherche (ref. D31-3159, D31-1162, D31-7055), in the framework of a project entitled “Caractérization et dynamique des fusarioses sur maïs en Région Wallonne.” BS acknowledges support by P.O.R. SARDEGNA F.S.E. 2007–2013—Obiettivo competitività regionale e occupazione, Asse IV Capitale umano, Linea di Attività l.3.1 (research project “Identification of natural and natural-like molecules inhibiting mycotoxin biosynthesis by *Fusaria* pathogenic on cereals”). UT thanks the Danish Directorate for Food, Fisheries and Agri Business grant FFS05-3 for financial support. The work of TY was financially supported by the Academy of Finland (no. 126917, 131957, 250904, 252162, 267188, and 266984), Olvi Foundation, Turku University Foundation, a CIMO travel grant to Taha Hussien, and the Nordic network project New Emerging Mycotoxins and Secondary Metabolites in Toxigenic Fungi of Northern Europe (project 090014), which was funded by the Nordic Research Board.

### Conflict of interest statement

The authors declare that the research was conducted in the absence of any commercial or financial relationships that could be construed as a potential conflict of interest.

## Supplementary material

Supplementary File 1 **Template for participants of the original initiative**.

Supplementary File 2 **Instruction to upload new dataset on the database and an output example for a given strain**.

Supplementary File 3 **(A) *Fusarium graminearum* and (B) *Fusarium culmorum* chemotype distribution within each participating country in % of all samples analyzed (n)**. 3-ADON, 3-acetyldeoxyniavlenol; 15-ADON, 15-acetyldeoxyniavlenol; NIV, nivalenol.

Supplementary Video 1 ***Fusarium graminearum* sampled during 6 years in Luxembourg**. Every year, at least 15 fields were sampled in different geographic locations in the country. Depending on the year, the species was more or less present. The NIV chemotype disappeared in the last 4 years of sampling in this species.
