# Supplementary material for: A European Database of Fusarium graminearum and F. culmorum Trichothecene Genotypes
Source: Front Microbiol. 2016 Apr 6;7:406. doi: 10.3389/fmicb.2016.00406 (PMC4821861; doi:10.3389/fmicb.2016.00406)

Supplementary file 2

How to upload a new dataset:

1. Register to the database selecting a username, a password and an institution. If the institution is not present, please add the information by creating a new institution.
2. Download the Excel template
3. Complete the Excel template with your data
4. Click on “Upload new dataset button”
5. Select the location of the edited file via the “browse” button and press “upload”
6. Wait for a confirmation via e-mail
7. The dataset will be validated and uploaded on the web

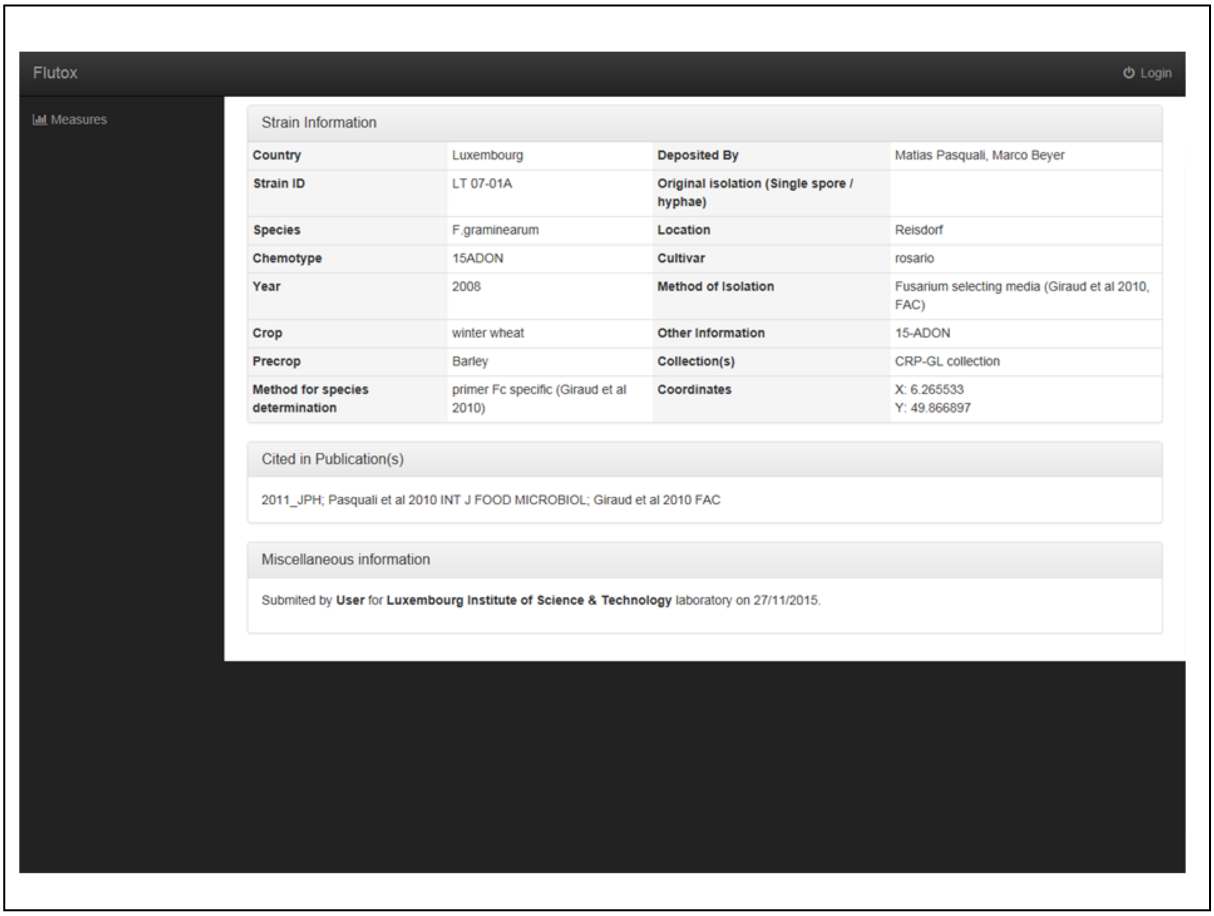

Supplement: Supplementary File 2 — Instruction to upload new dataset on the database and an output example for a given strain. [file Image2.PDF]
