# Supplementary material for: A European Database of Fusarium graminearum and F. culmorum Trichothecene Genotypes
Source: Front Microbiol. 2016 Apr 6;7:406. doi: 10.3389/fmicb.2016.00406 (PMC4821861; doi:10.3389/fmicb.2016.00406)
Supplement: Supplementary File 3 — (A) Fusarium graminearum and (B) Fusarium culmorum chemotype distribution within each participating country in % of all samples analyzed (n). 3-ADON, 3-acetyldeoxyniavlenol; 15-ADON, 15-acetyldeoxyniavlenol; NIV, nivalenol. [file Image3.PDF]

Zur optimalen Anzeige dieses PDF-Portfolios sollte es in Acrobat oder Adobe Reader ab Version X geöffnet werden.

Adobe Reader jetzt herunterladen
