# Supplementary material for: A European Database of Fusarium graminearum and F. culmorum Trichothecene Genotypes
Source: Front Microbiol. 2016 Apr 6;7:406. doi: 10.3389/fmicb.2016.00406 (PMC4821861; doi:10.3389/fmicb.2016.00406)
Supplement: Supplementary File 1 — Template for participants of the original initiative. [file Image1.pdf]

Supplementary file 1

Country	Participant	Isolate_ID	Species	Chemotype	Year of isolation	Single spore / single hyphae	Location: community, province, country	Crop_host	Cultivar	Previous crop	Method of isolation	Method used for species attribution (morphological	Other information (e.g. chemotype, toxins observed, virulence, …)	Deposited in culture colle	Cited in publication/s	Other remarks	x coordinate	y coordinate
Luxembourg	Pasquali_Beyer	LT10_27	Fculmorum	3ADON	2007	yes	Contern, Luxembourg	wheat	Aron	Wheat	Fusarium selecting media (Giraud et al 2010, FA	primer Fc specific (Giraud et al 2010)	chemotype determined with Ward et al 2002 (tri 12 polymorphism)	LIST fungal collection	Pasquali et al 2011_JPH; Pasquali et al 2010 INT J FOOD MICROBIOL; Giraud et al 2010 FAC		6.2263	49.5858
